# Crystal Chemistry and Thermoelectric Properties of Type-I Clathrate Ba_8_Ni_∼3.8_Si_*x*_Ge_42.2−*x*_ (*x* = 0, 10, 20, 42.2)

**DOI:** 10.3390/ma11060946

**Published:** 2018-06-04

**Authors:** Yue Dong, Xueyong Ding, Xinlin Yan, Long Zhang, Zhaohui Tang, Weiliang Chen, Peter Rogl, Silke Paschen

**Affiliations:** 1 School of Metallurgy, Northeastern University, Shenyang 110819, China; dong_y2014@163.com (Y.D.); tangzhaohui31@163.com (Z.T.); cookiechenwl@163.com (W.C.); 2 Institute of Solid State Physics, Vienna University of Technology, Wiedner Hauptstr. 8–10, 1040 Vienna, Austria; paschen@ifp.tuwien.ac.at; 3 State Key Laboratory of Metastable Materials Science and Technology, Yanshan University, Qinhuangdao 066004, China; lzhang@ysu.edu.cn; 4 Institute of Materials Chemistry and Research, University of Vienna, Währingerstr. 42, 1090 Vienna, Austria; peter.franz.rogl@univie.ac.at

**Keywords:** thermoelectric properties, crystal structure, clathrates, phase equilibrium

## Abstract

Thermoelectric materials are actively considered for waste heat recovery applications. To improve the heat to electricity conversion efficiency, fundamental understanding on composition, crystal structure, and interrelation with the thermoelectric properties is necessary. Here, we report the chemical and thermoelectric properties of type-I clathrates Ba${}_{8}$Ni${}_{3.8}$Si${}_{x}$Ge${}_{42.2-x}$ (*x* = 0, 10, 20, 42.2), to show that the Si substitution can retain the low lattice thermal conductivity as in pure Ge-based clathrates by adding defects (cage distortion) scattering and/or alloying effect, and the charge carrier concentration can be optimized and thus the electronic properties can be improved by tailoring the vacancy content. We demonstrate the vacancies in the pure Ge-based compound by Rietveld refinement, and possible vacancies in the quaternary compound by transport property measurements. We also show that, for intrinsic property studies in these compounds with such a complex crystal structure, a heat treatment for as cast alloys is necessary for phase purity and composition homogeneity. The highest ZT value of 0.19 at 550 ${}^{{}^{\circ}}C$ is reached in the compound with x=10.

## 1. Introduction

As representatives of phonon glass–electron crystal (PGEC) materials, intermetallic type-I clathrates have been attracting great attention for fundamental investigations and potential applications in the field of thermoelectricity [1,2,3,4,5,6,7,8,9,10]. The crystal structure of type-I clathrate contains covalently bonded frameworks with elements mainly from groups 13 and 14 in the periodic table, forming cages for metal atoms to fill [8,9,11]. The rattling of the metal atoms in these cages was generally considered as the root for the low lattice thermal conductivity in type-I clathrates [5,6,8], which benefits the high thermoelectric (TE) performance determined [1,12,13,14,15] by $ZT=S^{2}\sigma T/\left(\kappa_{e}+\kappa_{ph}\right)$. In this formula, *S* is the Seebeck coefficient (or thermal power), σ the electrical conductivity, *T* the absolute temperature, $\kappa_{e}$ and $\kappa_{ph}$ the electronic and phonon contributions to the total thermal conductivity κ in the material, respectively. Researchers have been focusing on adjusting these transport parameters to enhance the TE performance such as increasing *S* and σ or decreasing κ. However, it is challenging due to the inversion relations between these parameters [16]. Substitution/doping with transition metal (TM) elements for the framework atoms is usually effective to optimize the charge carrier concentration [8,9,11,17,18,19,20,21,22,23,24,25,26,27,28], which is frequently guided by the Zintl law because type-I clathrates are considered as Zintl compounds [8,9,11,29,30]. The substituted TM elements generally have different valences from the framework atoms. By accepting electrons donated by filled metal (e.g., Ba) in the cages, the TM element forms a similar electron configuration as the framework atoms (such as Si) and then bonds covalently with them. The residual electrons (or holes, in the case of a lack of bonding electrons) contribute to the transport properties in the system. The transport properties can then be tailored by changing the TM content. However, vacancies, which are typical in type-I clathrates (especially in Ge containing clathrates) [8,9,11], should be considered in the use of the Zintl law. They accept electrons and reduce the residual carrier concentrations. The content of vacancies in the crystal structure is generally difficult to control, as it depends on many factors such as the type of metals in the cages, the substitution level of a TM element for the framework, and the material synthesis process [8,9]. It is even still challenging to precisely define the quantity. On the other hand, the homogeneity of phase composition also influences the prediction of the charge carrier concentration by the Zintl law. A well designed sample preparation process should be performed to obtain perfect specimens for intrinsic physical property investigations.

So far, type-I clathrates with high ZT values are comprised of expensive elements such as Ga and Ge; for instance, Ba${}_{8}$Ga${}_{16}$Ge${}_{30}$ has a high value of 1.35 at 900 K [31]. For applications, effort has also been taken for clathrates with cheap elements such as TM, Si, and Al [32,33,34,35,36]. However, Si-based clathrates currently have low ZT values. Both electronic properties and thermal conductivity need to be optimized. A “cross-substitution” could improve these properties by more flexibly changing the electronic band structure and introducing more scattering centers [8,26,37,38,39,40,41,42,43,44,45,46,47]. It brings in the meantime interest for the fundamental investigation on chemical properties such as structural variations and stabilities.

To continue our study on multi-element clathrates, here we report the chemical and TE properties of type-I clathrate in the Ba-Ni-Ge-Si system. Systematic research has been performed on clathrates in the ternary Ba-Ni-Ge and Ba-Ni-Si systems, focusing on the phase equilibria (mostly the solid solution of Ni in the clathrate phase) [21,23,24,27,28,48], crystal structures [19,21,24,48], charge carrier concentration tuning [19,21,23,27,28,49], and other physical properties [21,28,48,49]. The solid solubilities of Ni in both Ge- and Si-based clathrates are less than around 4.2 at./f.u. (atom/formula unit) and vacancies exist in both crystal structures [21,27,28,49]. It is a rare case that vacancies exist in the framework in Si-based clathrates [9]. In the Ge-based Ni containing clathrates, the transport properties vary systematically with the Ni content. A metal–insulator transition can be observed in the Ni range between 3.8 and 4.2 at./f.u. [28,49]. A sign change in *S* can be seen when the Ni content is around 4.0 at./f.u. [28,49]. No p-type semiconductor can be seen in the Si-based clathrates [21,23,48]. In both systems, the compounds close to the Zintl compositions have promising TE properties [28,49]. Therefore, in our study, we focus on samples with a Ni content of 3.8 at./f.u. and combine both Si- and Ge-based ternary clathrates for quaternary ones, aiming to study the chemical and physical properties.

## 2. Results and Discussion

### 2.1. Phase Analysis

**Figure 1:**
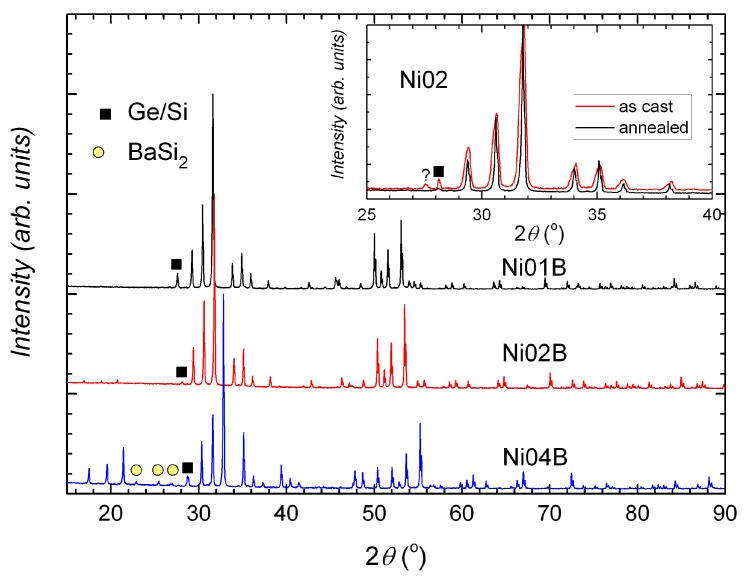
XPD patterns of annealed samples Ni01B, Ni02B, and Ni04B. Different tiny phases can be seen in the annealed samples. Inset: XPD patterns of Ni02A (as cast) and Ni02B (annealed). The annealed sample has very narrow peaks and a small amount of secondary phase (Si/Ge).

**Figure 2:**
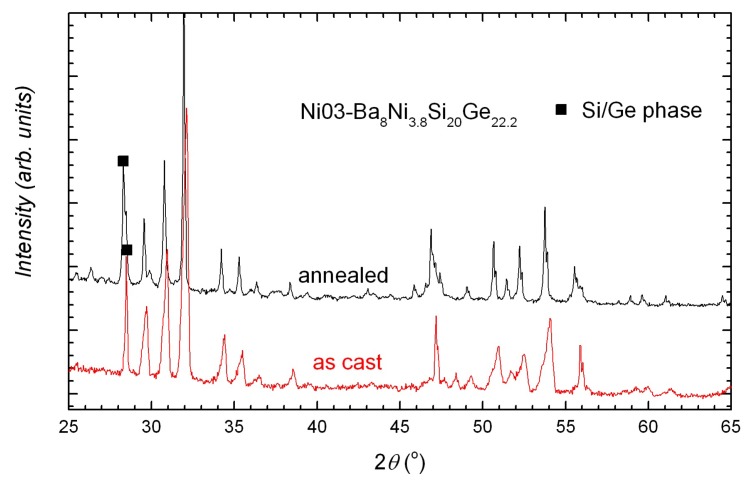
XPD patterns of Ni03A (as cast) and Ni03B (annealed). Narrow peaks can be seen in the annealed sample. Ni03B has more secondary phases than Ni03A.

In all samples, the main phase is, as expected, the type-I clathrate phase. The secondary phases depend on the nominal composition and the treatment state, which also determine the compositional homogeneity of the clathrate phase. Broadened peaks or peak splitting can be seen in the X-ray powder diffraction (XPD) patterns of the as cast samples and are more obvious in the quaternary compounds (Figure 1 inset and Figure 2). After annealing, the samples Ba${}_{8}$Ni${}_{3.8}$Si${}_{x}$Ge${}_{42.2-x}$ (*x* = 0, 10, 20, 42.2, denoted by Ni01B–Ni04B) are more homogeneous (Figure 1, reflected by very narrow peaks) and have less secondary phases (Figure 1 inset) (Ni03B has strangely more secondary phases (Figure 2)).

The inhomogeneity in as cast samples may indicate a sluggish kinetic factor for reaching the designed composition. This may be more serious in the Si/Ge-based quaternary compounds. The inhomogeneity in composition was confirmed by energy dispersive X-ray spectroscopy (EDX) measurements. These results indicate that an appropriate heat treatment is necessary for the phase homogeneity, especially in the multi-element systems. The sluggish kinetic factor could also be the reason for the compositional variation in single crystals grown by a floating zone technique [46].

**Figure 3:**
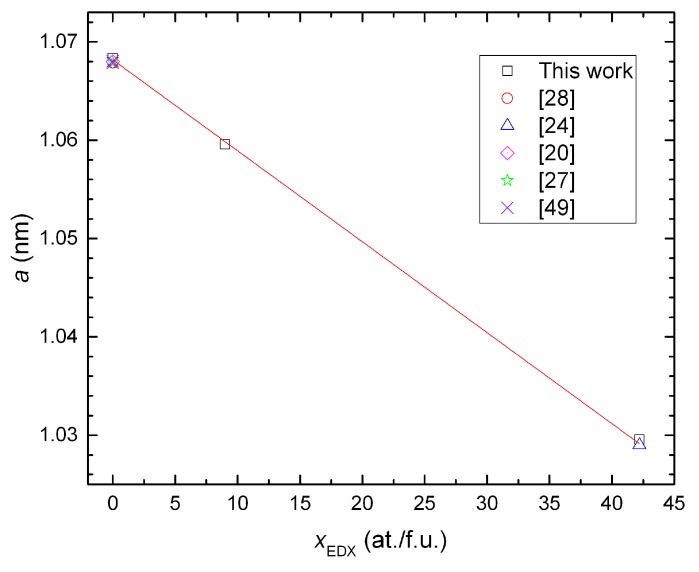
Lattice parameter *a* vs. the Si content in the clathrate phase $x_{\mathrm{EDX}}$. The lattice parameters of the ternary clathrates of similar compositions from the literature are included for comparison.

The lattice parameters evaluated from annealed samples, which are almost single-phase alloys, show a linear dependence with the Si content of the clathrate phase ($x_{\mathrm{EDX}}$ represents the composition measured by EDX and normalized by assuming 54 at./f.u.). This dependence basically follows Vegard’s law and reveals the mixture of Si and Ge atoms in the framework of the quaternary compound. We omitted the data from Ni03B because the large amount of secondary phases affect not only the contents of Si and Ge, but also the Ni content in the clathrate phase. The lattice parameters of the ternary clathrates are comparable with the literature data [20,24,27,28,49] (see Figure 3).

### 2.2. Crystal Chemistry

The structural data of Ni01B, Ni02B, and Ni04B were derived by Rietveld refinements with the XPD data at room temperature. An initial model of Ni atoms occupying the 6c site, Ba occupying the 2a and 6d sites, and Si/Ge occupying or sharing the framework sites 16i and 24k of the type-I clathrate (SG: Pm-3n) was used for the refinements. Due to the limited resolution of the lab X-ray resources and high backgrounds in the diffraction patterns, we did not try to refine the vacancies in the Si containing samples (Ni02B and Ni04B) and site-splitting at the 6*d* or 16*i* sites in all samples as in the literature [19,20,21,24]. In addition, because of the similar X-ray scattering factors of Ge and Ni, we fixed the Ni content according to the EDX measurements in both Ge containing samples (Ni01B and Ni02B). Thus, we could derive the vacancy content in Ni01B by assuming Ni, Ge and vacancies sharing the 6c site. By refining the Ge content at that site, we obtain a very small value of 0.13 at./f.u. of vacancies (Table 1), which is close to the reported data in a similar composition [20].

In the quaternary clathrate Ni02B, although the Si atoms can be found in all framework sites, most of them situate at the 16i sites. As mentioned, we cannot exclude that vacancies exist at the 6c site. The refinements gave the composition similar to the measured one by EDX, very good reliability factors, and reasonable temperature factors.

Surprisingly, we found that a very small amount of Ni atoms is necessary at the 24k site in order to obtain temperature factor similar to the other sites.

In all samples, the atomic parameter *x* at the 16*i*, *z* at the 24*k*, and the size of cages show systematic changes with the Si content. However, the atomic parameter *y* at the 24k site shows no systemic change (see Table 1). Nonlinear behavior for the atomic parameters was also reported in Ba${}_{8}$Cu${}_{5}$Si${}_{x}$Ge${}_{41-x}$ [26].

**Figure 4:**
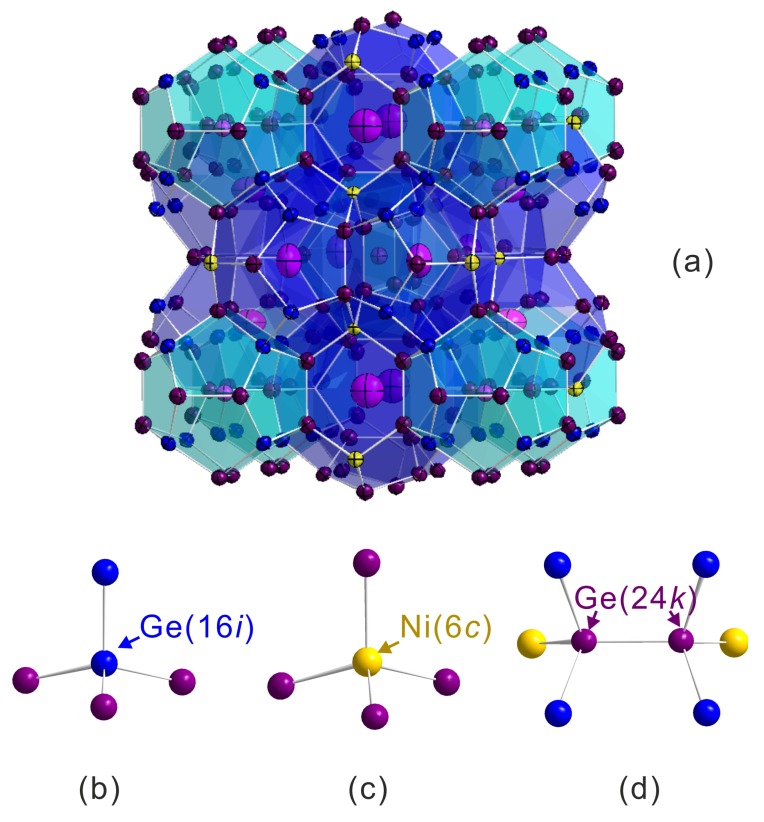
Crystal structure of type-I clathrate (**a**) and atomic environment (tetrahedral bonds) in different sites (**b**) Ge(16i); (**c**) Ni(6c); and (**d**) Ge(24k).

The interatomic distances are shown in Table 2. We show only relevant interatomic distances such as Ba atoms at the 2*a* and 6d sites to the framework atoms and atoms to the tetrahedrally bonded atoms in the framework as shown in Figure 4. To clarify the changes of interatomic distance induced by the Si substitution, we compared interatomic distances in the samples Ni01B and Ni02B (Table 2). From the distance difference Δd and reduction percentage $P_{i}$, we observe that the substitution shrinks both cages in a manner that the shrinkage in some direction is stronger than in others (e.g., Δd in the small cage). This indicates that the cages have been distorted by the Si substitution. On the other hand, we notice that the largest difference Δd (or reduction percentage $P_{i}$) is from the interatomic distance Ge(16i)-Ge(16i), which confirms that the Si atoms mainly locate at the 16*i* site in the Ni02B sample (see Table 1).

### 2.3. Thermoelectric Properties

In the present work, we show only the thermoelectric (TE) properties of hot pressed (HP) samples since the annealed samples are too brittle to obtain appropriate specimens for transport measurements. The XPD patterns of the HP samples are similar to the corresponding annealed samples (not shown); we thus consider all HP samples are almost phase-pure. For the Ni03B sample, after hot pressing, there is still a noticeable amount of secondary phases, thus we will not show the TE properties for comparison with the other samples.

**Figure 5:**
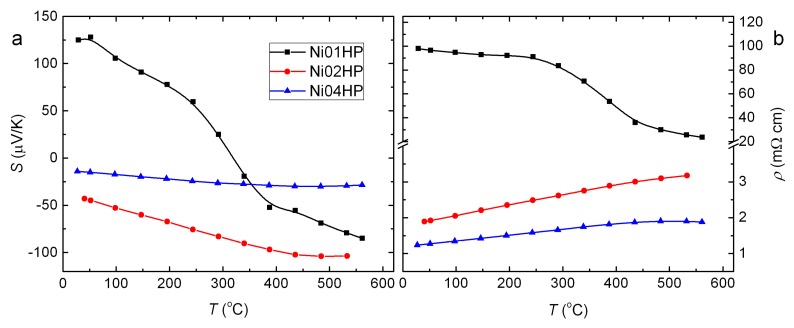
Temperature dependence of Seebeck coefficient (**a**) and electrical resistivity (**b**) for the series of hot pressed samples.

The Seebeck coefficient (*S*) of the samples Ni02HP and Ni04HP is negative, indicating that electrons are the major carriers (Figure 5a). A sign change is observed in Ni01HP in the measured temperature range. This may imply that the sample composition Ba${}_{8}$Ni${}_{3.8}$Ge${}_{42.2}$ is close to the critical composition for a metal–insulator transition. To understand the sign change, we estimate the carrier type and the charge carrier concentration by the Zintl law. Based on the expression [Ba${}^{+2}$]${}_{8}$[Ni${}^{-4}$]${}_{3.8}$[Ge${}^{0}$]${}_{42.2}$ with valences presented in superscripts [20], we calculate the carrier concentration by $n_{e}=16-3.8\times 4=0.8$ e${}^{-}$/f.u., which is not very small but suggest n-type behavior. Even including the vacancies of 0.13 at./f.u., each accepting four electrons, the calculated charge carrier concentration is 0.28 e${}^{-}$/f.u. However, the observed positive Seebeck coefficient below 300 ${}^{{}^{\circ}}C$ may indicate that the vacancy content estimated from the Rietveld refinement is too low, or the Ni content in the compound might be higher than the EDX value. The latter could be most likely since the reported composition for a sign change (from positive to negative) by increasing temperature is higher than 4.0 at./f.u. for the Ni content [28,49].

**Figure 6:**
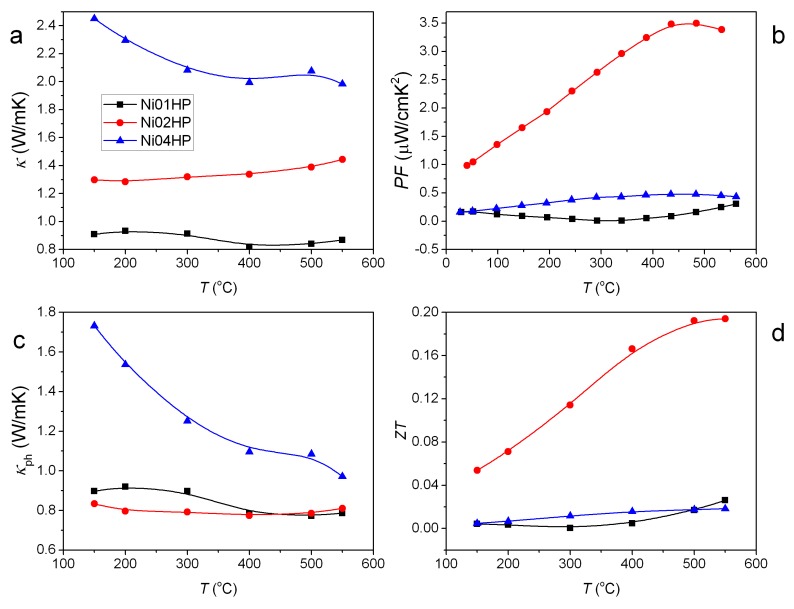
Temperature dependence of total thermal conductivity κ (**a**); power factor PF (**b**); lattice thermal conductivity (phonon contribution) $\kappa_{ph}$ (**c**); and ZT (**d**) for the series of hot pressed samples.

The different vacancy accommodation capability in Si- and Ge-based clathrates might provide a new strategy to improve the TE performance: by mixing Si- and Ge-based clathrates, we can adjust the vacancy content in the sample and thus improve electronic properties (Figure 6b). In the meantime, we can retain the low lattice thermal conductivity as in the Ge-based clathrates (Figure 6c) by adding defects (cage distortion) scattering and/or alloying effect [50]. The higher lattice thermal conductivity in the Si-based sample (Ni04HP) sample than the Ge-based sample (Ni01HP) is due to the lighter mass for Si. The vacancy filling by the Si substitution also can possibly improve the carrier mobility as clathrates in the Ba-Cu-Ga-Ge system [46], which benefits the reduced electrical resistivity and high TE performance. The strategy seems to be effective in the Ni-containing clathrates (Figure 6b–d). We expect that it would work also in other TM-element containing clathrates or even other Zintl compounds where vacancies can be tailored by element substitutions, even an isoelectronic element as Ge by Si.

The temperature dependence of ZT for all samples is shown in Figure 6d. Ni01HP has the lowest ZT value in all samples in most of the temperature range, mainly due to the highest electrical resistivity. The quaternary sample Ni02HP has the best ZT values. The highest ZT value is 0.19 at 550 ${}^{{}^{\circ}}C$.

## 3. Materials and Methods

High purity elements (more than 99.99 wt %) were used for preparing samples Ba${}_{8}$Ni${}_{3.8}$Si${}_{x}$Ge${}_{42.2-x}$ (*x* = 0, 10, 20, 42.2) (nominal composition) in an arc melting furnace. An excess of Ba (1.5 wt %) was added to balance the evaporation in the melting process. Each alloy was melted four times for homogeneity. The as cast samples are denoted by Ni01A to Ni04A for *x* changing from 0 to 42.2 in the nominal composition. The as cast samples were sealed in quartz tubes in vacuum and annealed at $800{\;}^{{}^{\circ}}C$ for 96 h in a muffle furnace prior to quenching in ice water. The annealed samples were then ball milled with process parameters: milling time: 4 h, energy mode: low energy, material of the container and balls: tungsten carbide, ball size: ϕ=1 cm, atmosphere: Ar, device: Pulverisette 5. The as milled powder was pressed at $800{\;}^{{}^{\circ}}C$ by hot pressing with duration of 2 h and pressure of 56 MPa at Ar atmosphere in a home-made device. The hot pressed samples have relative densities (ratio of measured density to theoretic density) around 95%.

The phase constituent in all samples was checked by X-ray powder diffraction (XPD) and scanning electron microscopy (SEM). XPD data were collected by X’Pert PRO (PANalytical B.V., Almelo, Netherlands) with Cu-K${}_{\alpha }$ radiation (λ(Cu-K${}_{\alpha 1})=1.54056$ Å, λ(Cu-K${}_{\alpha 2})=1.54443$ Å, no monochromator, $10^{{}^{\circ}}\leq 2\theta \leq 90^{{}^{\circ}}$, step: 0.002${}^{{}^{\circ}}$). The crystallographic data were determined from Rietveld refinement of the XPD patterns with the program FULLPROF [51]. The SEM experiments were performed by Zeiss Supra 55VP (Carl Zeiss AG, Oberkochen, Germany) coupled with energy dispersive X-ray spectroscopy (EDX, probe size: 1 μm, voltage: 20 kV).

The electrical resistivity and Seebeck coefficient were measured with a ZEM-3 (ULVAC-Riko, Kanagawa, Japan) in a temperature range between room temperature and $600{\;}^{{}^{\circ}}C$. The thermal conductivity was calculated by $\kappa =D_{t}C_{p}D$, where the thermal diffusivity $D_{t}$ was measured with a flash method in a Flashline-3000 (ANTER, Pittsburgh, PA, USA); the specific heat $C_{p}$ was measured in Flashline-3000 using a comparative procedure with NIST (National Institute of Standards and Technology) steel as reference; and the bulk density *D* was measured by the Archimedes method. The temperature range for the $D_{t}$ (and $C_{p}$) measurements is from $150{\;}^{{}^{\circ}}C$ to $600{\;}^{{}^{\circ}}C$, with a step of $50{\;}^{{}^{\circ}}C$. The density *D* in corresponding temperatures was calculated with the room temperature density and the thermal expansion coefficient, which referred to the similar compositions in the systems Ba-Ni-Ge/Si [52].

The lattice thermal conductivity $\kappa_{\mathrm{ph}}$ is calculated by subtracting the electronic contribution $\kappa_{e}$ from the total thermal conductivity $\kappa_{\mathrm{tot}}$ ($\kappa_{\mathrm{tot}}=\kappa_{\mathrm{tot}}-\kappa_{e}$). The electronic contribution $\kappa_{e}$ is estimated by the Wiedemann–Franz law $\kappa_{e}=L_{0}T/\rho$ with $L_{0}=2.44\times 10^{-8}$ (V/K)${}^{2}$ ($L_{0}$ basically depends on the Fermi level [53]).

The measurement uncertainties are 2% for the electrical resistivity and Seebeck coefficient and 7–10% for the thermal conductivity at high temperatures.

## 4. Conclusions

In summary, the chemical and TE properties of Ba${}_{8}$Ni${}_{3.8}$Si${}_{x}$Ge${}_{42.2-x}$ (*x* = 0, 10, 20, 42.2) have been investigated. An inhomogeneity in phase composition has been observed in all as cast samples and more obviously in quaternary compounds. Heat treatments can improve the phase homogeneity and reduce the secondary phases. Structural investigations revealed that vacancies exist in the pure Ge-based compound, and possibly also in the quaternary compound Ba${}_{8}$Ni${}_{3.8}$Si${}_{10}$Ge${}_{32.2}$, evidenced by the comparison of the transport properties with the pure Si-based compound. In this quaternary compound, although Si can be found in all framework sites, most of them are located at the 16i sites, which was further confirmed by analysing the interatomic distances. The Si substitution in the framework distorted both cages in the structure, which might be one of the reasons for the low lattice conductivity in the quaternary compound. Together with the improved power factor, which was attributed to an appropriate vacancy content and thus charge carrier concentration, we observed enhanced TE performance in the quaternary compound Ba${}_{8}$Ni${}_{3.8}$Si${}_{10}$Ge${}_{32.2}$. The highest ZT value of 0.19 has been reached at 550 ${}^{{}^{\circ}}C$. We expect that the strategy via changing the vacancy content to adjust the charge carrier concentration and concomitantly though distorting the cages to retain low thermal conductivity by means of a elemental substitution for high TE performance will also be effective in other Zintl cage compounds.

## Figures and Tables

**Table 1:** Structure data for annealed samples Ni01B, Ni02B, and Ni04B. The Ni content was fixed to the results from EDX. No vacancies were assumed at the 6c site. The unit for $B_{eq}$ is 10${}^{2}$(nm${}^{2}$).

Sample Code	Ni01B	Ni02B	Ni04B
Composition, EDX	Ba${}_{8}$Ni${}_{3.8}$Ge${}_{42.2}$	Ba${}_{8}$Ni${}_{3.4}$Si${}_{9.0}$Ge${}_{33.3}$	Ba${}_{8}$Ni${}_{3.5}$Si${}_{42.5}$
Composition, Refine	Ba${}_{8}$Ni${}_{3.8}$Ge${}_{41.9}$	Ba${}_{8}$Ni${}_{3.4}$Si${}_{9.2}$Ge${}_{33.4}$	Ba${}_{8}$Ni${}_{3.7}$Si${}_{42.3}$
Lattice parameter *a*, nm	1.06835(2)	1.05959(2)	1.02958(1)
$R_{F}=\sum \left|F_{o}-F_{c}\right|/\sum F_{o}$	0.057	0.045	0.052
$R_{I}=\sum \left|I_{o}-I_{c}\right|/\sum I_{o}$	0.058	0.062	0.069
Ba1, in 2a (0,0,0) $B_{eq}$	0.4(1)	0.5(1)	0.5(1)
Ba2, in 6d ($\frac{1}{4},0,\frac{1}{2}$) $B_{eq}$	2.6(1)	2.1(1)	1.55(5)
M1, in 6c ($\frac{1}{4},\frac{1}{2},0$), Occ.	3.8Ni + 2.07(5)Ge	3.4Ni + 2.2(1)Ge	3.4Ni + 2.6(1)Si
	+ 0.13□	+ 0.6Si	
$B_{eq}$ 10${}^{2}$ (nm${}^{2}$)	0.3(2)	0.8(1)	0.87(9)
M2 in 16i (x,x,x), *x*	0.1835(2)	0.1843(2)	0.1862(2)
Occ.	16Ge	11.3(1)Ge + 4.7Si	16Si
$B_{eq}$	0.7(1)	0.4(9)	0.84(9)
M3 in 24k (0,y,z), y,z	0.1225(2), 0.3154(3)	0.1232(2), 0.3140(2)	0.1217(2), 0.3095(3)
$B_{eq}$	0.3(1)	0.7(2)	0.4(1)
Occ.	24Ge	20.1(1)Ge + 3.9Si	23.7Si + 0.3(1)Ni
Size of cages, $V_{s}$, $V_{l}$ in Å${}^{3}$	127.3, 167.7	123.6, 163.3	110.2, 149.7
2nd phases	Ge	Ge(Si)	Si + BaSi${}_{2}$

**Table 2:** Selected interatomic distances (Å, error bar is ∼0.0006 Å) for Ni01B, Ni02B, and Ni04B, as well as the distance difference between Ni01B and Ni02B, Δd = $d_{i}$(Ni01B)-$d_{i}$(Ni02B), where *i* denotes an interatomic distance such as Ba(2a)–8Ge(16i) and the reduced percentage (%), $P_{i}=\Delta d/d_{i}$(Ni01B).

*A*	n*B*	$d_{i}$(Ni01B)	$d_{i}$(Ni02B)	$d_{i}$(Ni04B)	Δd	$P_{i}$
Ba(2a)	−8Ge(16i)	3.3965	3.3829	3.3211	0.0136	0.4
	−12Ge(24k)	3.6148	3.5740	3.4241	0.0408	1.1
Ba(6*d*)	−8Ge(24k)	3.5687	3.5504	3.4702	0.0183	0.5
	−4Ni(4c)	3.7772	3.7462	3.6401	0.0310	0.8
	−8Ge(16i)	3.9723	3.9353	3.8136	0.0370	0.9
	−4Ge(24k)	4.0930	4.0500	3.9423	0.0430	1.0
Ni(6c)	−4Ge(24k)	2.3969	2.3852	2.3648	0.0117	0.5
Ge(16i)	−1Ge(16i)	2.4592	2.4104	2.2744	0.0488	2.0
	−3Ge(24k)	2.5008	2.4744	2.3931	0.0264	1.0
Ge(24k)	−1Ni(6c)	2.3971	2.3854	2.3648	0.0117	0.5
	−2Ge(16i)	2.5008	2.4744	2.3931	0.0264	1.1
	−1Ge(24k)	2.6475	2.6102	2.5068	0.0373	1.4
